# Optimal complementary feeding practice and associated factors among children in Konso Zone, South Ethiopia

**DOI:** 10.3389/fnut.2025.1568887

**Published:** 2025-07-08

**Authors:** Meseret Girma Abate, Zelalem Tafese Wondimagegne, Tefera Belachew

**Affiliations:** ^1^School of Nutrition, Food Science and Technology, Hawassa University, Hawassa, Ethiopia; ^2^Department of Nutrition and Dietetics, Faculty of Public Health, Jimma University, Jimma, Ethiopia

**Keywords:** optimal complementary feeding, meal frequency, dietary diversity, Konso Zone, South Ethiopia

## Abstract

**Introduction:**

Despite the critical importance of complementary feeding, a significant number of young children in developing countries have suboptimal complementary feeding practices (OCFPs). After 6 months, a nutrient-dense, varied diet containing fruits and vegetables is crucial to complement breastfeeding. These appropriate complementary feeding practices have the potential to prevent all deaths among children. There were evidence gaps on OCFPs among children in the study area. Therefore, this study was conducted to fill this gap in order to design context-specific intervention.

**Method:**

A community-based cross-sectional study was conducted in Konso Zone, in South Ethiopia, among 337 randomly selected mothers having children 6–23 months of age. Data were collected using a pretested interviewer-administered questionnaire using the Kobo Toolbox and exported to SPSS Version 25 for cleaning and analysis. A multivariable binary logistic regression model was used to determine independent predictors of OCFP of mothers. Variables with a *p*-value < 0.05 at a 95% confidence interval (CI) were considered statistically significant in the final model.

**Results:**

The overall prevalence of OCFP was 14.8% (95% CI: 11.80%, 19.10%). The practices of timely initiation of complementary feeding, minimum meal frequency, and minimum dietary diversity (MDD) were 63.20%, 92.60%, and 20.50%, respectively. Mothers who were in advanced age (>35 years) (AOR = 3.32, 95% CL: 1.59, 6.95), exchanged food items from the market (AOR = 2.23, 95% CL: 1.03, 4.77), and had accessibility and availability of fruit and vegetables (AOR = 4.16, 95% CL: 1.83, 9.43) were independent predictors of OCFP.

**Conclusion:**

The findings indicate that a significantly low proportion of children met the minimum World Health Organization (WHO) complementary feeding recommendations. However, meal frequency showed relatively better adherence. Only one-fifth of young children achieved MDD, while more than two-thirds began complementary feeding earlier than the recommended 6-month threshold. To improve complementary feeding practices, cost-effective interventions such as increasing access to fruits and vegetables and encouraging mothers to trade homegrown food items in local markets to diversify their children’s diets could be beneficial. Additionally, targeted efforts should focus on enhancing key complementary feeding indicators of meal frequency, timely introduction of complementary foods, and achieving MDD to improve children’s nutritional needs.

## 1 Introduction

Adequate nutrition throughout infancy is vital for a child’s optimal growth and development later in life (1). The first 2 years are critical for physical and cognitive development, yet infants are vulnerable to developmental insults caused by inadequate nutrition and diseases (1). Optimal complementary feeding practice entails fulfilling dietary diversity, meal frequency, early breastfeeding initiation, exclusive breastfeeding up to 6 months, and continued breastfeeding until the age of two (2, 3).

At 6 months, an infant’s energy and nutrient demand increases. Complementary feeding should be timely initiated, nutritionally adequate, and diverse to meet the child’s growing needs while continuing breastfeeding (4, 5). However, many infants and children do not receive adequate or appropriate feeding. Globally, only 44% of infants are exclusively breastfed for 6 months, 29.4% receive the minimum dietary diversity (MDD), and 52.2% receive the minimum required meal frequency (6).

In Ethiopia, only 59% of children aged 0–6 months were exclusively breastfed, and just 8% of infants aged 6–23 months received complementary foods while continuing breastfeeding. The 2019 Ethiopian Demographic and Health Survey (EDHS) reported MDD at 14%, minimum meal frequency (MMF) at 55%, and minimum acceptable diet (MAD) at 11% (7, 8).

A study conducted in the Somali regional state of Ethiopia revealed that 49.40% had optimal complementary feeding practices (OCFPs), with 57% initiating complementary feeding between 6 and 8 months, 52% achieving minimal meal frequency, and 45% attaining dietary diversity (9). Similarly, a study conducted in the Jimma Zone of West Ethiopia found the overall proportion of OCFP was 9.40% (10).

Several factors influence optimal breastfeeding and complementary feeding, including maternal knowledge gaps, perceived breast milk insufficiency, excessive workload, limited partner support, and food price inflation (11, 12).

Childhood malnutrition remains a pressing issue in low- and middle-income countries, driven by inadequate nutrition and poor feeding practices in infancy (13). Recognizing the importance of adequate and appropriate nutrition for child health and survival, the Ethiopian government implements health extension programs at the household and community level to improve optimal complementary feeding for children aged 6–23 months (14).

Despite child health being a priority under Ethiopia’s healthcare development program (15), the national prevalence of appropriate complementary feeding practices among children aged 6–23 months was extremely low and is below international standards. There was evidence of gaps in OCFPs, and related factors are crucial for prioritizing, designing, and implementing intervention programs to reduce childhood undernutrition. Thus, this study aimed to assess OCFPs and associated factors among children aged 6–23 months in the Konzo Zone, South Ethiopia.

## 2 Materials and methods

### 2.1 Study settings and target population

A community-based cross-sectional study was undertaken among 337 mothers who had children aged 6–23 months in Konso Zone, Southern Ethiopia, from May 10 to June 30, 2024. In this zone there are three districts, which consist of three to five health posts and three to eight kebeles. The Zone is characterized by rugged and rocky highlands cut by deep valleys. The altitude of the area is between 501 and 2,000 m above sea level, and the main agro-ecological divisions of Konso are 70% lowland and 30% tropical midland (16).

### 2.2 Sample size determination and procedures

The sample size was determined by using the single population proportion estimation formula. To calculate the actual sample size, a 30% proportion from a previous related study on OCFP (17), 95% confidence level, 5% margin of error, and a 10% non-response rate were considered. The final sample size comprised 354 children aged 6 to 23 months paired with their mothers.

Two districts were selected randomly from a total of three districts in the Konso Zone, South Ethiopia. In the case of Ethiopia, at least one health post was built for one to two kebeles for the provision of primary healthcare. From each district, one kebele that had a nearby health post was selected purposively. Then a census was conducted in two kebeles who had nearby health posts (the lowest administrative level in the case of Ethiopia) to register eligible households who had children aged 6–23 months. After getting the number of mothers who had children 6–23 months of age from all selected kebeles. The number of children assigned is based on the proportion of the size of the eligible population at all levels. Finally, the required number of mothers who had children in each kebele was selected randomly for interview.

### 2.3 Data collection tools and measurement

A structured and pretested questionnaire was used to collect data on socio-demographic characteristics (18), dietary practices, and related characteristics. Data on dietary diversity and meal frequency data were collected by using a 24-h recall method (19). Mothers were asked to recall all food items given to their children in the past 24 h before the day of the survey. According to the World Health Organization (WHO) 2021 guidelines, MDD for children 6–23 months was collected by using eight food groups: (1) grains, roots, or tubers; (2) legumes and nuts; (3) vitamin A-rich FV; (4) other fruits or vegetables; (5) flesh foods (meat, poultry, fish, organ meat); (6) eggs; (7) dairy products (milk, yogurt, cheese); and (8) breast milk (5). In addition, the household food security was collected using the Household Food Insecurity Access Scale (HFIAS) to measure the degree of food security level in the past 30 days (20).

Exchange of food items grown on their own land, including sorghum, maize, and moringa leaf cabbage. After selling this product, mothers purchase food items like avocados and some cereals like barley and wheat from local markets that are not cultivated in the area for preparing complementary diets (21). Additional data was collected to measure household accessibility and availability of fruit and vegetables using questionnaires that measure the existence of different fruits and vegetables at home during the data collection period, either from their own agricultural cultivation or purchasing from local markets (22, 23). After pre-testing, the questionnaire was loaded onto Kobo Toolbox for face-to-face interviews at household level.

### 2.4 Data quality control

Data quality was ensured in all phases of the research activities from methodological design to data analysis. The questionnaire was prepared in English first and then translated into Amharic for consistency for interviews. A pre-test was conducted on 5% of the total sample of the population of similar profiles but not included in the main study. Four health professionals who had previous data collection experience were recruited. Before actual data collection, training was provided to data collectors on the sampling procedures and interview techniques of the study. The data collectors and supervisors were also proficient in the local language.

### 2.5 Operational measurement

Optimal complementary feeding practice: quantified using a composite indicator comprising three of the WHO core infant child feeding indicators that relate closely to complementary feeding. These are the timely introduction of solid complementary feeding, MDD, and minimum meal frequency. If a child fulfilled the above three criteria, classify them as having received optimal complementary food (5).

Minimum dietary diversity score: ranges from 0 to 8 diversified food groups. In this study, children who received five or more from eight food groups within 24 h were considered to have MDD (5).

Timely introduction of complementary feeding: measured as the proportion of infants 6–23 months of age who received solid, semi-solid, or soft foods at 6 months (5).

Minimum meal frequency (MMF): is the proportion of children who received at least the recommended minimum meal frequency appropriate for age in the last 24 h prior to the survey: at least 2 feeding times daily for infants aged 6–8 months and at least 3 times daily for young children aged 9–23 months within 24 h (5).

Food-insecure household: a household that experiences one of the three levels of food insecurity conditions mildly, moderately, or severely food insecure or access conditions in the past 4 weeks is categorized as food insecure (20).

Exchange of food items from the market: measured using a questionnaire to assess household selling of food items produced by their own agricultural cultivation, such as sorghum, maize, and moringa leaf cabbage. After selling this agricultural own cultivation product, mothers purchase from the local market food items like avocado and some cereals like barley and wheat that are not cultivated in the area (21).

Accessibility and availability of fruit and vegetables: accessibility and availability of fruits and vegetables were measured using questionnaires that measured the household existence of different fruits and vegetables, either from their own agricultural cultivation or from purchases at local markets during 24-h recalls (22, 23).

Nutrition education: measured using questionnaires that address whether a child’s mother gets nutrition education about breast and complementary feeding during pregnancy, at the time of birth and in the postnatal period from health professionals and health extension workers. Then mother nutrition education status is categorized as getting nutrition education (Yes) if mothers get nutrition education by health professionals and health extension workers at health and community settings and not getting nutrition education (No) if those who did not get any nutrition education about child breast and complementary feeding by health professionals and health extension workers are categorized as not getting nutrition education (No) (24, 25).

### 2.6 Data analysis

Data was exported from Kobo Toolbox to an Excel sheet and SPSS version 26 for analysis. A descriptive analysis was conducted to characterize the data using frequencies, percentages, means, and standard deviations. Socio-demographic variables of the child, such as sex and age of the child, and the child’s mother’s education and occupation, were categorized based on related literature and biological classification (18, 19). The wealth index of households was measured using principal component analysis (26). Kaiser–Meyer–Olkin (KMO; value of *p* > 0.05) and Bartlett’s test of sphericity (value of *p* < 0.05) were used to check the adequacy of the sample-to-factors ratio. The existence of a correlation between each item was determined for each step of factor analysis (27, 28). In PCA, factor scores were generated using variables with a commonality value greater than 0.5. Finally, the household’s wealth index was calculated as non-dummy variables were divided into three categories: highest, medium, and lowest household wealth index (27).

Multicollinearity was assessed by conducting variance inflation factor (29). After testing, the multicollinearity variance inflation factor of variables was below 10 (VIF < 10) and the SE was <2. This result indicated there was no existence of multicollinearity among variable which entered to multivariable logistic regression model. Then binary logistic regression analysis was used to identify factors associated with OCFPs. In addition, before conducting multivariable logistic regression analysis model fitness was tested by the Hosmer–Lemeshow test (*p* = 0.638). All variables with a *p*-value less than 0.25 in the binary logistic regression analysis were included in the multivariable logistic regression analysis model. Variables with a *p*-value < 0.05 and AOR with a 95% confidence interval (CI) were considered statistically significant.

### 2.7 Ethical consideration

Ethical clearance was obtained from the Hawassa University College of Medicine and Health Sciences institutional review board (Ref. No: IRB/045/16). Informed verbal assent was obtained from all mothers instead of their young children after a clear explanation about the purpose of the study. Then a permission letter was obtained from the Konso Zone Health Office. All the study participants were reassured that they would be anonymous. Names or any personal identifiers were not recorded.

## 3 Results

### 3.1 Socio-demographic characteristics of young children and women

A total of 337 women were involved in the study, giving a response rate of 95.19%. The mean age of young children was 13.86 + 5.207 months. In terms of sex of child, 52.50% of children were males and 47.50% were females. More than half of mother had two (61.70%) and above children and the rest 38.30% had one child. More than half of the mothers (58.5%) were aged between 25 and 34 years. Additionally, 75.4% of the respondents had no formal education (Table 1).

**TABLE 1:** Socio-demographic characteristics of children’s and mothers in the Konso Zone, South Ethiopia, 2024.

Variables	Category	Frequency	Percentage
Sex	Male	177	52.5
	Female	160	47.5
	≤1 under five child	129	38.2
	≥2 under five child	208	61.7
Age of child	From 6 to 8 months	90	26.7
	From 9 to 11 months	41	12.2
	From 12 to 23 months	206	61.1
Age of mother	18–24 years	71	21.1
	25–34 years	197	58.5
	Above 35 years	69	20.5
Educational status of mother	No formal education	254	75.4
	Elementary education	66	19.6
	Secondary and above	17	5
Occupation of mother	Farmer	311	92.3
	Government worker	26	7.7
Religion of mother	Orthodox	30	8.9
	Protestant	307	91.1
Wealth index	Poor	135	40.1
	Medium	67	19.9
	Rich	135	40.1

### 3.2 Optimal complementary feeding practices

**FIGURE 1:**
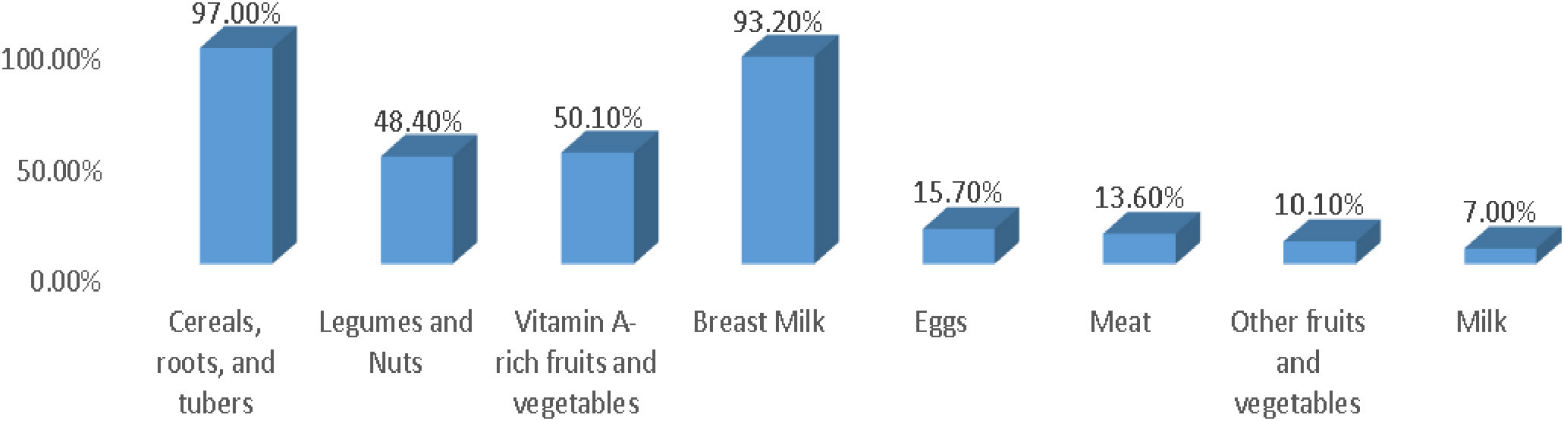
Proportion of children who consumed the dietary diversity components during the 24 h prior to the day of data collection in 6–23 months of children in the Konso Zone, Southern Ethiopia, 2025.

The prevalence of OCFP was 14.80% (95% CI: 11.80%, 19.10%) of children. Of which, 92.60% had adequate meal frequency, 20.50% had good dietary diversity practice, and 63.20% had started complementary food at 6 months. Among all interviewed mothers, 20.50% fed their child five or more food items, and the rest, 79.5%, fed <5 food items within 24 h preceding the survey. The dominant food groups fed to their child were cereals, roots, and tubers, which accounted for 79.7%, but low feeding practice was observed in animal source foods (milk, meats, and eggs), and other fruits and vegetables were 7.7%, 13.6%, 15.7%, and 10.1%, respectively (Figure 1).

**FIGURE 2:**
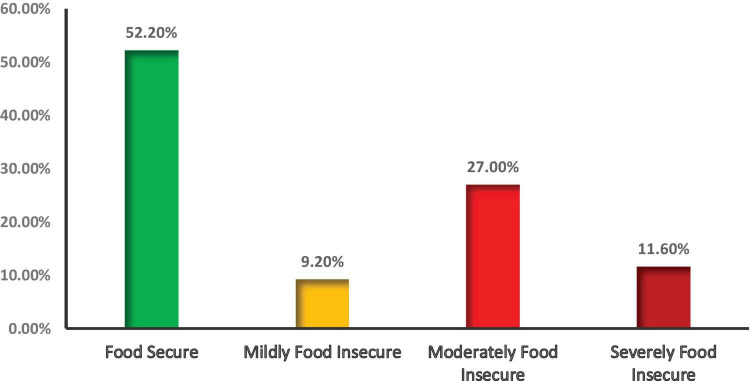
Household food security status of mothers who have children 6–23 months old, Konso Zone, in Southern Ethiopia, 2025.

Above half (52.20%) of the households were food secure, while the rest were food insecure with different severity: mildly insecure (9.2%), moderately insecure (27.00%), and severely food insecure (11.60%) (Figure 2). This figure indicates that half of the households in the study setting may face suboptimal dietary practices resulting either from limited access to food supply. This limited access to food supply affected dietary diversity and meal frequency.

### 3.3 Factors associated with optimal complementary feeding practices

After checking the basic assumption of the binary logistic regression model, multiple binary logistic regression models were fitted to identify significant factors. Mothers who were of advanced age (>35 years) (AOR = 3.2; 95% CL: 1.59, 6.95, *p*-value 0.001), exchange of food items from the market (AOR = 2.23; 95% CL: 1.03, 4.77, *p*-value 0.042), and accessibility and availability of fruit and vegetables (AOR = 4.16; 95% CL: 1.83, 9.43, *p*-value 0.001) were significantly associated with OCFPs (Table 2).

**TABLE 2:** Multivariable logistic regression predicting the likelihood of optimal complementary feeding practices among 6–23 months of children in the Konso Zone, Southern Ethiopia, 2025.

Variables		Optimal complementary feeding practices		COR with 95% CL			AOR	95% CI		*p*-Values
		**Yes**	**No**		**LCL**	**UCL**		**LCL**	**UCL**	
Age of child	From 6 to 8 months	14 (28.0%)	76 (26.5%)		1			1		
	From 9 to 11 months	10 (20.0%)	31 (10.8%)	0.57	0.23	1.42	0.59	0.22	1.61	0.058
	From 12 to 23 months	26 (52.0%)	180 (62.7%)	1.27	0.63	2.58	2.16	0.97	4.78	0.505
Sex of child	Male	27 (54.0%)	150 (52.3%)	1.07	0.59	1.96	0.59	0.22	1.61	0.505
	Female	23 (46.0%)	137 (47.7%)		1			1		
Age of mothers	18–34 years	31 (62.0%)	237 (82.6%)		1			1		0.001**
	≥35 years	19 (38.0%)	50 (17.4%)	2.91	1.52	5.55	3.32	1.59	6.95	
Wealth index	Poor	19 (38.0%)	116 (40.4%)					1		
	Medium	5 (10.0%)	62 (21.6%)	1.46	0.76	2.78	0.97	0.47	1.99	0.931
	Rich	26 (52.0%)	109 (38.0%)	2.96	1.08	8.09	2.47	0.844	7.24	0.099
Number of under five children	Having one child	21 (42.0%)	108 (37.6%)		1			1		
	Having more than one children	29 (58.0%)	179 (62.4%)	1.2	0.65	2.21	1.24	0.63	2.46	0.524
Getting nutrition education	Yes	17 (34.0%)	66 (23.0%)	1.73	0.9	3.29	1.73	0.83	3.61	0.147
	No	33 (66.0%)	221 (77.0%)		1			1		
Exchange of food items from market	Yes	14 (28.0%)	36 (12.5%)	2.71	1.33	5.51	2.23	1.03	4.77	0.042**
	No	36 (72.0%)	251 (87.5%)		1			1		
Accessibility and availability of fruit and vegetables	Yes	9 (18.0%)	131 (45.6%)	3.83	1.79	8.16	4.16	1.83	9.43	0.001**
	No	41 (82.0%)	156 (54.4%)		1			1		
Food security status	Food security	30 (60.0%)	177 (61.7%)	0.93	0.51	1.72	0.67	0.34	1.34	0.261
	Food insecurity	20 (40.0%)	110 (38.3%)		1			1		

AOR, adjusted odd ratio; LCL, lower confidence limit; UCL, upper confidence limit. **Significant factors (*p*-value < 0.05).

## 4 Discussion

The results showed that OCFP was 14.80% (95% CI: 11.80%, 19.10%). This finding signifies that OCFP is very low as compared to the WHO and other related studies finding (2, 4). This low OCFP contributed to inadequate nutrient intake for young children and directly exposed them to undernutrition (4, 30). This finding was similar with the study conducted in Ghana, and North Eastern Ethiopia which accounts 15.70% and 18.10% (30, 31). However, the finding of this study was higher than the reported prevalence of OCFPs in Southern Ethiopia was 8.60% (19), and 9.40% in Jimma Zone of Southwest Ethiopia (6), and secondary data analysis of the 2019 mini EDHS (9.70%) (20). This discrepancy might be due to variations in the socioeconomic status, cultural practices, mothers’ perceptions, and their awareness of what and when to introduce proper complementary foods to their children, as well as the belief that children at this age are unable to absorb solid foods (2, 19). Additionally, variations in health facility services, such as the availability of health education and advice on complementary feeding practices during the first 2 years of life, may also contribute.

The feeding practice of dietary diversity, where mothers fed their children five or more food items from eight food groups within the previous 24 h, was 20.50%. Almost one in five children did not get diversified food items, which is below the recommended level. This finding was in agreement with the study finding from Sidama Zone, South Ethiopia, which accounts for 20.40% (32), and was higher than the national prevalence of Ethiopia and Southwest Ethiopia, which were 14.00% and 15.20%, respectively (7, 10). However, the finding of this study was lower than the study findings in the Somali region in Eastern Ethiopia, which was 49.4% (9). This difference might be due to variation in food item availability and accessibility across different areas of Ethiopia. Significant geographic variation in fruit and vegetable consumption was observed throughout Ethiopia’s regions (33). In the current study setting, there is limited agricultural production of diversified food items, as the community produces dominantly cereals. Even though the community had access to animal source foods, mainly eggs, mothers/caregivers sold it to the local market instead of feeding their child. This leads to complementary food for children being cereal-based and monotonous.

In addition, the previous studies measured the MDD of children 6–23 months of age who consumed at least four foods out of seven defined food groups, while we used at least five out of eight defined food groups during the previous 24 h (9). Hence, the use of four out of seven defined food groups may overestimate the prevalence. Furthermore, the variation might be due to differences in the cultural and socioeconomic status of the study population, low maternal literacy, and poor dietary diversity promotion activities.

The prevalence of timely initiation of complementary feeding at 6 months in this study was 63.2%, while 36.80% started before 6 months. This finding was lower than the related studies done in various settings, such as the Soro district in Southwest Ethiopia, the Sidama Zone, and Gonder, Northwest Ethiopia, which reported prevalence of initiation of complementary feeding practices of 34.30%, 34.00%, and 47.30%, respectively (32, 34, 35). However, the finding of this study was higher than the study conducted in North East Ethiopia, which was 71.90% (36). This might be due to sociodemographic differences, including the low level of maternal education in our settings; almost three-fourths (75.40%) of mothers have no formal education. In addition, mothers in this study setting engaged in agricultural farming activities outside their home. Due to this role of women in farming, they stay far from their home after 2–3 months of postnatal periods and early complementary food is provided in place of breast milk. This brings early initiation of complementary feeding practice before 6 months as compared to other settings.

The result of this study revealed that the proportion of children who consumed complementary foods at least the minimum meal frequency during the prior day of the survey was found to be 92.60%. This was higher than findings from Southern Ethiopia (53.7%) (37), and Jimma in Southwest Ethiopia (64.10%) (10). This could be attributed to differences in the study period, socio-demographic characteristics, and the role of health development armies in nutrition-sensitive activities. Improved facility access and increased maternal contact with healthcare workers focusing on antenatal, postnatal, and child care education may also have promoted meal frequency feeding practices.

Mothers who had mothers who were of advanced age (>35 years) were three times more likely to practice optimal complementary feeding. This finding was supported by another study, which concluded that mother’s age was a predictor for complementary feeding practices because older mothers were more experienced and knowledgeable in taking care of their children compared to their younger and less knowledgeable counterparts (38). Older mothers may have better experience with complementary feeding practices as compared to young mothers.

Mothers who had an exchange practice of food items from the local market were two times more likely to practice optimal complementary feeding. This finding was in line with a study conducted in Ghana; improving market access could be important for promoting dietary diversification (39, 40). This may be food availability and access in local markets can promote mothers’ purchasing power or exchange of different food items from the market (41). Further evidence showed a consistent positive association between access to markets and dietary diversity (42). In addition, improved access to markets can influence household dietary diversity, consumption expenditure, and food security through multiple pathways. Market access can increase smallholder farmers’ income through decreased transaction costs and improve diet quality (43).

Mothers with access to fruits and vegetables were four times more likely to practice optimal complementary feeding for their children. The availability and accessibility of a variety of foods in both the market and household empower mothers to directly feed their young children and indirectly purchase food items for them. This contributes to improved overall complementary feeding practice (23, 40, 44). In addition, farm production diversity is positively associated with dietary diversity and directly enhances complementary feeding practices (23).

## 5 Strength and limitation of the study

This study comprehensively evaluates the minimum WHO complementary feeding recommendation indicators. As a limitation, mothers may not fully recall the food items provided for children. Seasonal variation is one of the limitations, even though the data collection period can represent the semi-harvest and cultivation season of the local area, but post-harvest seasonal data was not collected. To minimize this, probing was implemented to recall food items fed to children within 24 h.

## 6 Conclusion

The findings indicate that a significantly low proportion of children met the minimum WHO complementary feeding recommendations. However, meal frequency showed relatively better adherence. Only one-fifth of young children achieved MDD, while more than two-thirds began complementary feeding earlier than the recommended 6-month threshold. To improve complementary feeding practices, cost-effective interventions such as increasing access to fruits and vegetables and encouraging mothers to trade home grown food items in local markets to diversify their children’s diets could be beneficial. Additionally, targeted efforts should focus on enhancing key complementary feeding indicators meal frequency, timely introduction of complementary foods, and achieving MDD to better meet children’s nutritional needs.

## Data Availability

The original contributions presented in this study are included in this article/supplementary material, further inquiries can be directed to the corresponding author.
