# Correction: Optimal complementary feeding practice and associated factors among children in Konso Zone, South Ethiopia

**DOI:** 10.3389/fnut.2025.1667567

**Published:** 2025-08-29

**Authors:** Meseret Girma Abate, Zelalem Tafese Wondimagegne, Tefera Belachew

**Affiliations:** ^1^School of Nutrition, Food Science and Technology, Hawassa University, Hawassa, Ethiopia; ^2^Department of Nutrition and Dietetics, Faculty of Public Health, Jimma University, Jimma, Ethiopia

**Keywords:** optimal complementary feeding, meal frequency, dietary diversity, Konso Zone, South Ethiopia

In the published article, there were errors in the **Affiliations**. The author, Meseret Girma Abate, was incorrectly assigned to “College of Medicine and Health Sciences, Arba Minch University, Arba Minch, Ethiopia” and “School of Nutrition, Food Science and Technology, Hawassa University, Hawassa, Ethiopia”. The correct assigned affiliation should have been written as, “School of Nutrition, Food Science and Technology, Hawassa University, Hawassa, Ethiopia.” The author, Tefera Belachew, was incorrectly assigned to “College of Medicine and Health Sciences, Arba Minch University, Arba Minch, Ethiopia” and “Faculty of Public Health, Jimma University, Jimma, Ethiopia.” The correct assigned affiliation should have been written as, “Department of Nutrition and Dietetics, Faculty of Public Health, Jimma University, Jimma, Ethiopia.”

The original article has been updated.

